# CSN6, a subunit of the COP9 signalosome, is involved in early response to iron deficiency in *Oryza sativa*

**DOI:** 10.1038/srep25485

**Published:** 2016-05-03

**Authors:** Song Tan, Fang Liu, Xiao-Xi Pan, Yue-Peng Zang, Fei Jin, Wei-Xi Zu, Xiao-Ting Qi, Wei Xiao, Li-Ping Yin

**Affiliations:** 1 College of Life Science, Capital Normal University, Beijing 100048, China; 2 Department of Microbiology and Immunology, University of Saskatchewan, Saskatoon, SK, S7N 5E5, Canada

## Abstract

The COP9 signalosome (CSN) plays an important role in proteasome-mediated degradation by regulating CUL1 rubylation of the SCF ligase and is involved in many crucial biological processes. Here, we demonstrate a link between IDEF1 accumulation and the decline in COP9 derubylation activity in response to iron deficiency (-Fe) in rice (*Oryza sativa*). *CSN6* expression is rapidly down-regulated during Fe depletion, contributing to reduced CSN activity, as judged by *CSN5* and *CUL1* expression, indicating CSN6 is involved in the early stage response of -Fe. In contrast to *CSN6*, the IDEF1 protein and expression of several iron uptake/utilisation-related genes are increased in response to -Fe. Thus, we constructed *CSN6* transgenic sense and antisense lines and found that experimental depletion of CSN6 results in accumulation of the IDEF1 protein and up-regulation of several iron uptake/utilisation-related genes. Furthermore, IDEF1 can be decorated with K48-linked polyubiquitin and degraded via the 26S proteasome. Accumulated IDEF1 in antisense lines led to increased chlorophyll and Fe content in seedlings during -Fe. Collectively, the cellular CSN6 level is decreased during early stages of -Fe to ensure the rapid accumulation of IDEF1, which in turn up-regulates several iron uptake/utilisation-related genes to help overcome -Fe stress in rice.

The COP9 signalosome (CSN) is a multisubunit protein complex conserved among diverged organisms, including fission yeast, fruit fly, Arabidopsis, human, and probably blue-green algae^1^,^2^,^3^,^4^. It is believed to localise to the nucleus and is involved in many crucial biological processes, including regulation of the ubiquitin-proteasome degradation system, cell cycle, DNA repair, and plant responses to light and hormones^5^. CSN functions to remove the ubiquitin-like protein RUB1 (Related to Ubiquitin 1) from the cullin subunit of the Cullin-RING Ligase (CRL) family of E3 complexes, but it can also bind derubylated CRLs and maintain them in an inactive state^5^,^6^,^7^. The cycles of rubylation/derubylation of the cullin are essential for maintaining an optimal pool of active E3 complexes in proteasome-mediated degradation^6^,^8^. Therefore, CSN-mediated derubylation of the cullin subunit is important for the progress of proteasome-mediated regulation. CSN is composed of eight subunits (CSNs): six with the PCI (Proteasome, COP9 signalosome, eukaryotic Initiation factor) domain and two with the MPN (MOV34, PAD N-terminal) domain including CSN5 and CSN6. Among these subunits, the derubylation activity of the COP9 signalosome is mainly embedded within the MPN domain of CSN5^9^. Furthermore, derubylation relies on COP9 signalosome stability and integrity, and loss of function of CSN5, CSN6, CSN7, or CSN8 destabilises several other CSNs in Arabidopsis^10^. Every subunit has their special function to ensure COP9 activity. The binding of the rubylated equivalent of CRL, neddylated CRL, to CSN is sensed by CSN4 and communicated to CSN5 with the assistance of CSN6, resulting in activation of the derubylase/deneddylase^11^. CSN6, the other MPN subunit, acts as a linker between CSN5 and the whole complex^12^. Suppression of *CSN6A* in Arabidopsis exhibits diverse developmental defects, including defects in homeotic organ transformation, symmetric body organisation, and organ boundary definition^13^. Therefore, the CSN6 subunit seems to play a critical role in both complex stability and in regulation of holocomplex function.

Iron is one of the essential mineral nutrients required for plant growth and development. Because of low solubility in aerobic and alkaline conditions^14^, plants have acquired different mechanisms to ensure iron absorption under low availability. These strategies include strategy I and strategy II, found in non-graminaceous monocots and dicots and in graminaceous monocots, respectively^15^. In strategy I, iron is transported directly by Iron-Regulated Transporters (IRT)^16^,^17^ from the rhizosphere after reduction by ferric chelate reductase. Whereas in strategy II, or the chelation strategy, graminaceous plants secrete Fe chelators to form soluble Fe (III) complexes, which are transported by yellow stripe/yellow stripe-like (YSL) transporters into roots. So far, rice is the only plant described to use the combined strategy mechanism, which has all features of a strategy II plant (phytosiderophores (PS) release through TOM1/OsZIFL4 and Fe (III)-PS uptake through OsYSL15, the YS1 orthologue^18^,^19^) and some features of a strategy I plant (Fe^2+^ uptake using IRT transporters)^20^. In studies using rice grown under iron deficiency (-Fe), many components, including the PS and iron transporters, such as MAs (the mugineic acid family), IRT1, and YSL15 are up-regulated. IRT1 and YSL15 are two iron transporters with different iron substrates, e.g. Fe^2+^ and Fe (III)-PS^18^,^21^. Many iron uptake/utilisation-related genes, such as *IRO2* and *IRT1*, contain the iron deficiency-inducible *cis*-acting element, IDE1^22^. The transcription factor IDEF1 binds to IDE1^23^. During the first day of -Fe, defined as the early stage of -Fe, a majority of known iron uptake/utilisation-related genes, such as *IRO2*, *YSL15,* and *IRT1* are positively regulated by IDEF1, and therefore IDEF1 is essential for the early response to iron deficiency^24^. IDEF1 has unique histidine-asparagine repeats flanked by proline-rich regions. This characteristic region can directly bind several divalent metals, and the metal-binding signal is then transmitted to downstream pathways. However, the mechanism of regulating the stability and activity of IDEF1 is still unknown. In this report, we present evidence that IDEF1 is regulated by proteasome-mediated degradation and CSN6 is involved in this regulation during the onset of -Fe via the derubylation function of the COP9 signalosome. CSN6’s down-regulation at the early stage of -Fe contributes to IDEF1 accumulation. We have thus identified a new mechanism of IDEF1 regulation under -Fe conditions.

## Results

### *CSN6* is down-regulated during the early stage of iron deficiency in rice

**Figure 1:**
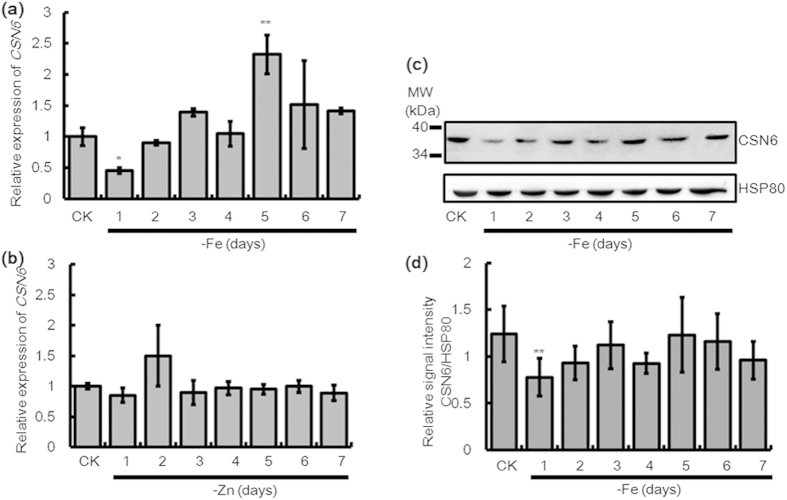
Relative expression change of *CSN6* at the mRNA and protein levels during -Fe. Change in the expression level of *CSN6* following growth under 7 days of (**a**) -Fe and (**b**) -Zn in 14-day-old WT seedlings by qRT-PCR. Relative transcript levels are compared with those in WT seedlings cultured on +Fe or +Zn (shown “CK”). (**c)** Immunoblot analysis of CSN6 protein accumulation patterns following growth under 7 days of -Fe in 14-day-old WT seedlings using an anti-CSN6 antibody. Equal protein loading was confirmed by immunoblotting with an antibody against rice HSP80. (**d)** The histogram represents quantification of the CSN6 bands by Image J. CSN6 protein levels are expressed as a ratio of CSN6 to HSP80. Error bars indicate SD (n = 3). Asterisks indicate P < 0.05 (*) and P < 0.01 (**) in Student’s t test analysis.

We screened a cDNA microarray of rice roots after five days of growth in an iron-free MS solution (-Fe)^25^ and found that the *CSN6* transcript accumulated in response to -Fe. Reverse transcription-PCR (RT-PCR) confirmed the up-regulation of *CSN6* after 5 days of -Fe, but its transcription also happened to decrease before five days of -Fe (data not shown). To further examine the *CSN6* expression pattern under -Fe, we performed time-course quantitative real time-PCR (qRT-PCR) analysis in wild-type (WT) rice plants. Seedlings were grown for two weeks in an MS solution with full nutrition (100 μM Fe, 30 μM Zn) and then transferred to a Fe-deficient (-Fe) or Zn-deficient (-Zn) condition for one to seven days. The qRT-PCR results also showed an increase in *CSN6* expression after five days of growth in the Fe-deficient condition. In addition, remarkably, the expression of *CSN6* in the -Fe plants after the first day initially decreased to 0.4-fold than that of the +Fe control (CK), but then increased to its normal level after day two in -Fe (Fig. 1a). In contrast to Fe deficiency, the expression of *CSN6* was not affected in response to Zn deficiency (Fig. 1b). We also evaluated the effect of -Fe on CSN6 protein level by immunoblot analysis using an anti-CSN6 antibody. In protein extracts from 14-day-old WT seedlings, CSN6 protein levels decreased after day one of -Fe, then began to rise in day two (Fig. 1c,d), and remained constant afterward. These observations suggest that *CSN6* is down-regulated at the mRNA level by -Fe in the first day, the early stage of -Fe, which affects the accumulation of CSN6 protein. Thus, we focused further studies mainly on the early stage of effective -Fe response.

### Activity of COP9 complex declines and IDEF1 accumulates in response to iron deficiency

**Figure 2:**
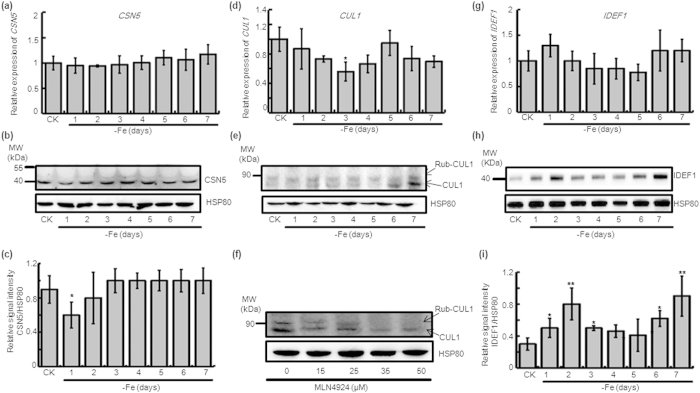
Relative expression levels of CSN5, CUL1, and IDEF1 in WT during iron-limited conditions. Changes in the expression level of *CSN5* following growth under 7 days of -Fe in 14-day-old WT seedlings by (**a**) qRT-PCR and (**b**) immunoblotting using an anti-AtCSN5 antibody, and (**c**) quantification of the CSN5 band using Image J. Changes in the expression level of *CUL1* following growth 7 days of -Fe in 14-day-old WT seedlings by (**d**) qRT-PCR and (**e**) immunoblotting using an anti-CUL1 antibody. (**f)** Immunoblot with an anti-CUL1 antibody on protein extracts from 14-day-old rice seedlings treated with 7d MLN4924. Changes in the expression level of *IDEF1* following growth under 7 days of -Fe in 14-day-old WT seedlings by (**g**) qRT-PCR and (**h**) immunoblotting using an anti-OsIDEF1 antibody, and (**i**) quantification of the IDEF1 band using Image J. Relative transcript levels were compared with those in WT seedlings cultured on +Fe (shown “CK”). Values shown are as the mean with SD (n = 3, *P < 0.05, **P < 0.01, Student’s t-test analysis). Protein levels are expressed as a ratio of CSN5 or IDEF1 to HSP80 and compared with the value for non-treated seedlings. Equal protein loading was confirmed by immunoblotting with an antibody against HSP80 in rice.

CSN serves an essential role in a myriad of cellular processes through the isopeptidase activity of its CSN5 subunit. CSN5 alone is inactive due to an auto-inhibited conformation of its catalytic domain, and the association of CSN5 with the CSN6 MPN (Mpr1/Pad1 N-terminal) domain activates its isopeptidase activity^26^. As CSN6 was down-regulated in response to iron deficiency, we investigated the isopeptidase activity of COP9 by examining CSN5 content. The *CSN5* transcript level remained constant during -Fe (Fig. 2a), but its protein level was decreased in day one of -Fe (Fig. 2b,c), coincident with CSN6. Since CSN functions in the derubylation of CRL, derubylation activity is also an indicator of COP9 activity. In response to -Fe, the *CUL1* mRNA level fluctuated during the early stages and only in day three of -Fe did it decrease (Fig. 2d). Detecting CUL1 by using an anti-CUL1 antibody showed two bands, rubylated CUL1 and native CUL1. The amount of native CUL1 protein also fluctuated in response to -Fe and was undetectable both in day one and day five of -Fe (Fig. 2e). Considering the stability of *CUL1* at the mRNA level and the decrease of the native band of CUL1 protein in day one, we concluded that the cause of this phenomenon is the decreased derubylation activity of COP9. These results indicate that COP9 activity is also reduced at early stage of -Fe.

MLN4924, a versatile and specific neddylation/rubylation inhibitor, has been regarded as a useful tool to examine the role of NEDD8- and CRL-dependent processes in a wide range of animal and plant species^27^,^28^. Rice seedlings treated with 35 μM of MLN4924 for seven days abolished the presence of the CUL1 rubylation modification (Fig. 2f). This indicates that MLN4924 could inhibit CUL1 rubylation in rice, and the band above CUL1 in Fig. 2e is rubylated CUL1, and rubylation can regulate the content of CUL1 and rubylated CUL1.

As CSN6 and CSN5 respond to -Fe by affecting the derubylation activity of COP9, we chose IDEF1 to further research the plant’s response to iron deficiency. IDEF1 is a transcription factor involved in early response to -Fe in rice^29^ and the expression of *IDEF1* was not affected in response to Fe deficiency (Fig. 2g). However, we found the protein level of IDEF1 began to accumulate during the first day of -Fe, and its levels continued to increase until day two (Fig. 2h,i). At day three, the amount of IDEF1 protein slightly decreased until day five (Fig. 2i). The increase of IDEF1 and decreased activity of COP9 caused by a declined in CSN6 shows that there is a negative relation between IDEF1 and CSN6.

### Up-regulation of iron utilisation-related genes in response to iron deficiency

**Figure 3:**
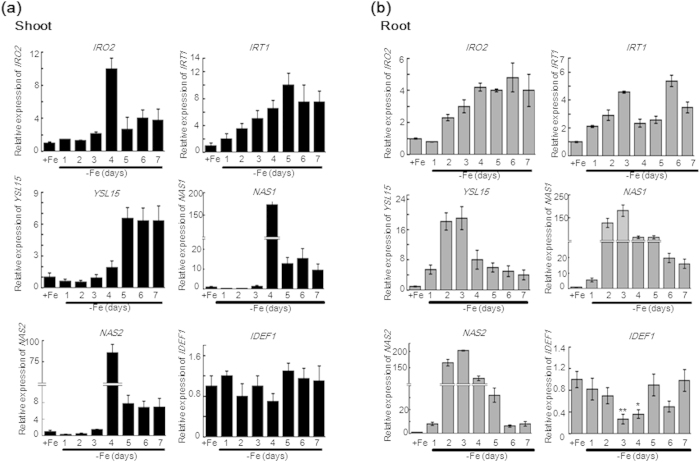
Quantitative analysis of typical iron utilisation-related genes during one to seven days of -Fe in WT plants. A qRT-PCR assay showing the temporal expression patterns of *IRO2*, *IRT1*, *YSL15*, *NAS1*, *NAS2,* and *IDEF1* in 14-day-old WT seedling shoots (**a**) and roots (**b**) after 7 days of -Fe. Relative transcript levels are compared with that in WT seedlings cultured on +Fe. Error bars indicate SD (n = 3). Asterisks indicate P < 0.05 (*) and P < 0.01 (**) in Student’s t test analysis.

The rice transcription factor IDEF1 plays a crucial role in regulating iron deficiency-induced genes involved in iron homeostasis. Consistent with previous reports, genes such as *IRO2*, *IRT1*, *YSL15*, *NAS1*, and *NAS2* were significantly up-regulated in WT seedling shoots (Fig. 3a) and roots (Fig. 3b) in -Fe. These genes were initially up-regulated in roots at day one of -Fe, and the genes in the shoots were up-regulated after three days of -Fe. In contrast to these iron utilisation-related genes, IDEF1 in shoots and roots exhibited a constitutive expression pattern in mRNA levels at early stages of -Fe (within 48-h) consistent with previous reports^29^. Post-transcriptional control of transcription factor activation is emerging as a key feature of several physiological processes in plants^30^. Our results indicate this level of control also exists to regulate IDEF1 at the protein level.

The basic helix-loop-helix (bHLH) transcription factor FIT with the four subgroup Ib bHLH proteins bHLH038, bHLH039, bHLH100, or bHLH101 represents the most upstream regulator in iron-deficiency signalling pathway of strategy I plants^31^. IDEF1 plays an analogous role in strategy II plants, with both proteins interacting with many downstream factors^23^. FIT protein stability under iron deficiency is regulated by 26S proteasome-dependent degradation^32^, and COP9 is involved in regulation of the ubiquitin-proteasome degradation system^5^,^33^. Considering the decreased activity of COP9 by down-regulation of CSN6 and increased protein accumulation of IDEF1 at the early stage of -Fe, we hypothesis the existence of a regulatory mechanism of IDEF1 protein degradation by CSN6.

### Detection and expression analysis of sense and antisense *CSN6* transgenic rice

**Figure 4:**
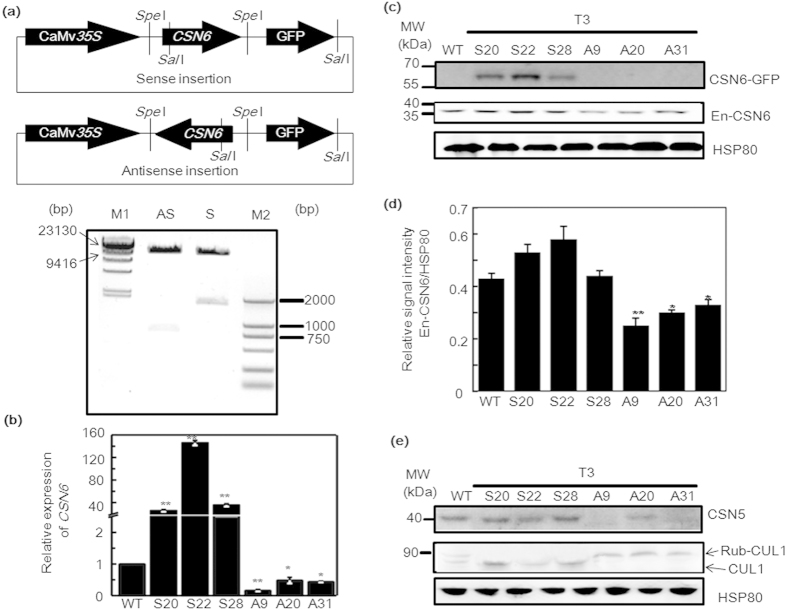
Construction and identification of *CSN6* sense (S) and antisense (A) transgenic rice lines. **(a)** Using *Spe*I sites, *CSN6* was cloned into pCAMBIA1302 with the cauliflower mosaic virus (CaMV) 35S promoter at the 3′-upstream end and GFP at the 5′-downstream end. Vectors were digested with *Sal*I (present either at the *CSN6* sequence or vector sequence) and run on an agarose gel to screen the sense and antisense recombinants. M1 represents the *Hind*III-digested λ phage DNA marker; M2 represents the DL2000 DNA marker. (**b**) Three sense (S) and three antisense (A) *CSN6* transgenic rice lines were chosen to determine the relative expression levels of *CSN6* by real-time PCR. Total RNA was extracted from 14-day-old *CSN6* transgenic rice seedlings. (**c**) Protein was extracted from 14-day-old *CSN6* transgenic rice seedlings and subjected to SDS-PAGE and immunoblot analysis. In sense lines, the exogenous CSN6::GFP fusion protein was detected using anti-GFP antibody. The endogenous CSN6 (En-CSN6) protein in sense and antisense lines was detected using an anti-CSN6 antibody. HSP80 was used as a loading control. (**d**) The histogram represents quantification of the endogenous CSN6 band using Image J. Protein levels are expressed as a ratio of CSN6 to HSP80. Error bars indicate SD (n = 3). Asterisks indicate P < 0.05 (*) and P < 0.01 (**) in Student’s t test analysis. (**e**) Expressions analysis of CSN5 and CUL1 in *CSN6* sense (S) and antisense (A) transgenic rice lines. Proteins were extracted from 14-day-old WT and T3 generations of *CSN6* transgenic rice seedlings and analysed using anti-AtCSN5 and anti-CUL1 antibodies, respectively. Equal protein loading was confirmed by immunoblotting with an antibody against HSP80 in rice.

To further investigate the effect of CSN6 on IDEF1 in the -Fe response, we examined *csn6* null mutants. However, all available Arabidopsis COP9 signalosome mutants exhibit severely retarded growth at the early seedling stage and are lethal after seedling development^13^. To circumvent this problem, *CSN6* transgenic rice lines were generated by introducing 35S promoter-driven sense and antisense *CSN6* cDNA into rice (Fig. 4a). Several positive transgenic strains of T1, T2, and T3 generations were obtained and identified. We selected three T3 sense (S) and antisense (A) transgenic lines, which were confirmed by qRT-PCR and immunoblot analysis. qRT-PCR analysis revealed that the relative expression levels of *CSN6* was markedly increased in lines S20, S22, and S28, and suppressed in lines A9, A20, and A31 (Fig. 4b). In immunoblot analysis of the *CSN6*, the exogenous CSN6::GFP fusion protein was detected in sense lines S20, S22, and S28 using an anti-GFP antibody (Fig. 4c). A decrease in endogenous CSN6 protein levels was observed in antisense lines A9, A20, and A31 using the anti-CSN6 antibody (Fig. 4c,d).

CSN6 is a subunit of the COP9 signalosome and links the derubylation catalytic subunit CSN5 to the holocomplex^12^. At*CSN6A* transgenic sense and antisense plants exhibit diverse developmental defects in Arabidopsis^13^. However, the exact function of CSN6 has not been elucidated so far in rice. The rice genome contains only one copy of both *CSN5* and *CSN6*. We examined the accumulation of CSN5 protein in both WT and *CSN6* transgenic plants by immunoblotting with an anti-AtCSN5 antibody (Fig. 4e). Compared with WT, *CSN6* antisense lines exhibited decreased CSN5 protein accumulation. In Arabidopsis, it has been shown that total depletion of CSN5, CSN6, or other subunits of the COP9 signalosome results in complete loss of derubylation activity and accumulation of rubylated cullin1, cullin3, and cullin4^10^. To examine the derubylation activity of the COP9 signalosome, we compared the rubylated/derubylated ratio of CUL1 in both WT and *CSN6* transgenic plants. As expected in *CSN6* antisense lines, decreased CSN6 protein levels resulted in increased levels of rubylated forms of CUL1 (Fig. 4e, Rub-CUL1). In contrast, increased CSN6 in the sense lines led to enhanced derubylation activity, and thus only presented the native band of CUL1 (Fig. 4e, CUL1). These results confirm that a reduction in the function of CSN6 leads to destabilisation of the CSN5 subunit, and therefore impairs the derubylation activity of the whole COP9 signalosome.

### Accumulation of high-molecular-weight IDEF1 in antisense *CSN6* transgenic rice

**Figure 5:**
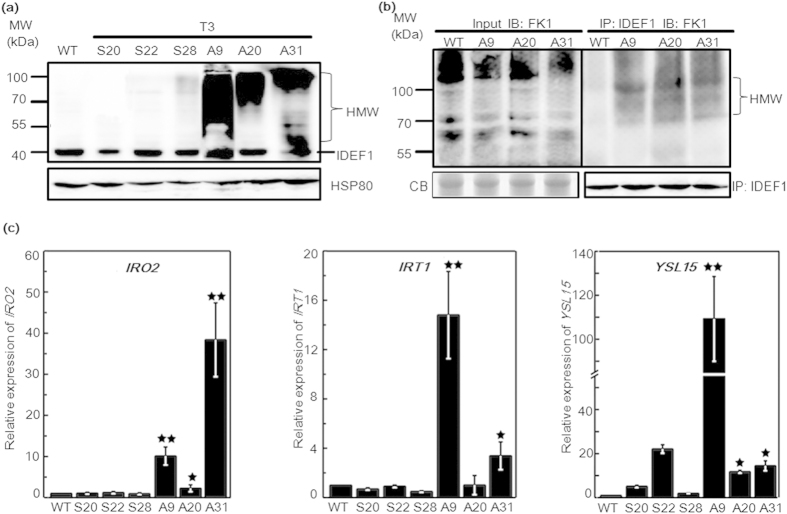
Quantitative analysis of IDEF1 and typical iron utilisation-related genes in *CSN6* transgenic plants. **(a)** IDEF1 protein analysis in *CSN6* transgenic rice. Proteins were extracted from 14-day-old WT and T3 generations of *CSN6* transgenic seedlings. Immunoblot analysis of native IDEF1 and modified IDEF1 (the high-molecular-weight, HMW) was performed with an anti-IDEF1 antibody in WT and *CSN6* transgenic plants (S and A). Equal protein loading was confirmed by immunoblotting with an antibody against HSP80. (**b**) Western blot analysis of the polyubiquitinated IDEF1 in *CSN6* antisense lines. Total proteins of 14-day-old T3 *CSN6* antisense lines and WT seedlings were immunoprecipitated by anti-IDEF1 antibody (IP: IDEF1) and detected by an anti-Ub (FK1) antibody (IB: FK1). IB, immunoblotting; IP, immunoprecipitation. Arrows indicate polyubiquitinated IDEF1. Equal protein loading was confirmed by CB (Coomassie Blue stain). Western blot using the anti-IDEF1 antibody was used as a loading control for the input of the IP. **(c)** qRT-PCR analysis of typical iron utilisation-related genes (*IRO2*, *IRT1,* and *YSL15*) in 14-day-old *CSN6* transgenic seedlings. Values represent means with standard deviations (n = 3, P < 0.05 (*), P < 0.01 (**) in Student’s t-test analysis).

IDEF1, a transcription factor that belongs to the ABI3/VP1 family, is ubiquitously expressed in roots, leaves, flowers, and seeds throughout the life cycle of rice^23^,^24^. In the work presented here, up-regulated IDEF1 can be detected under -Fe condition (Fig. 2i) and this may be caused by CSN6 reduction. To better understand the role of CSN6 in IDEF1 degradation, we examined the signal of IDEF1 in *CSN6* sense and antisense lines. High-molecular-weight IDEF1 (HMW-IDEF1) protein accumulated in all three *CSN6* antisense lines, and this accumulation was barely observed in sense lines and WT (Fig. 5a). We then asked whether the modified bands were ubiquitinated IDEF1. To test this we immunoprecipitated IDEF1 from antisense and wild type lines using an anti-IDEF1 antibody (Fig. 5b). Western blot analyses of IDEF1 immunoprecipitates with an anti-Ub antibody FK1, which recognises polyubiquitin chains, showed that ubiquitinated IDEF1 was much more abundant in antisense lines than in the wild type (Fig. 5b). Hence, experimental reduction of *CSN6* expression resulted in an accumulation of polyubiquitinated IDEF1. Most notably, native IDEF1 was also increased in antisense lines A9 and A20 (Fig. 5a). Zhang *et al*.^34^ has reported the native IDEF1 accumulated after MG132 treatment, an 26S proteasome inhibitor. This result indicates de-ubiquitination of IDEF1 coexists simultaneously with ubiquitination. Then to address the biological significance of IDEF1 accumulation in *CSN6* antisense lines, we measured IDEF1 target gene expression in the *CSN6* transgenic lines. Compared with WT and *CSN6* sense lines, accumulation of the positive regulator IDEF1 in the *CSN6* antisense lines, as such accumulated native IDEF1, resulted in up-regulation of several genes involved in iron uptake, such as *IRO2*, *IRT1*, and *YSL15* (Fig. 5c).

CSN functions as an isopeptidase that removes the ubiquitin-like activator RUB from CRLs, but it can also bind derubylated CRLs and maintain them in an inactive state. Therefore, less active CSN leads to increasing SCF ubiquitination activity in antisense line or at early stage of iron deficiency, and results in more ubiquitinated IDEF1. Meanwhile, disrupted CSN activity leads to ubiquitinated IDEF1 inaccessible to proteasome-mediated degradation and thus accumulated ubiquitinated IDEF1 in antisense lines. In antisense lines, native IDEF1 was also increase (Fig. 5a) and expression of *IRO2* was also remarkable increased (Fig. 5c). Iron utilisation-related genes were up-regulated mainly by native IDEF1^15^,^29^, especially like *IRT1* and *YSL15* in A9 and A20 (Fig. 5c). Furthermore, the native IDEF1 accumulated under MG132 treatment^34^. It will be further studied whether the ubiquitinated IDEF1 may help increase active IDEF1 level through de-ubiquitination or not. At last, the increase of active IDEF1 up-regulates its target genes to help overcome the -Fe stress. Taken together, the accumulation of IDEF1 protein in response to -Fe or in the *CSN6* antisense lines indicates that IDEF1 degradation is regulated by CSN6.

### IDEF1 can be polyubiquitinated and degradated via the proteasome pathway

**Figure 6:**
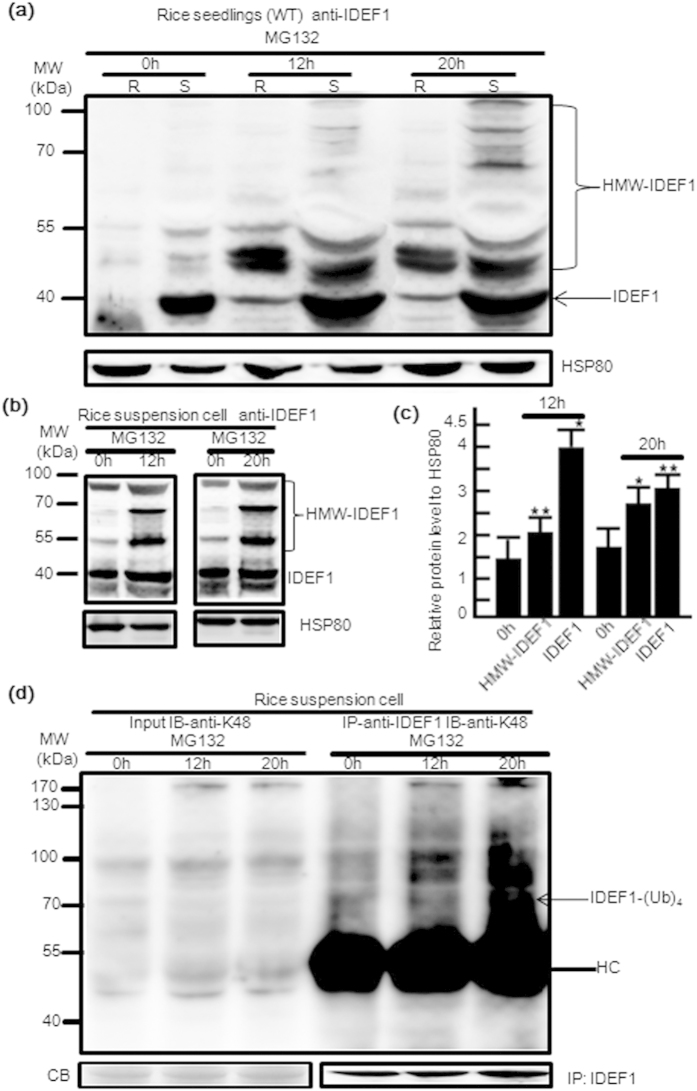
IDEF1 undergoes proteasome-mediated degradation. (**a**) Immunoblot analysis of IDEF1 protein in rice seedlings treated with MG132. The 14-day-old WT rice roots (R) and shoots (S) treated with DMSO (0 h) or 40 μM MG132 for 12 h and 20 h were used for protein extraction. Immunoblot analysis of native IDEF1 and modified IDEF1 (the high-molecular-weight, HMW) with an anti-IDEF1 antibody and HSP80 as loading controls. The arrow indicates unmodified IDEF1. (**b**) Immunoblot analysis of IDEF1 protein in rice suspension cells treated with 40 μM MG132 for 12 h and 20 h. (**c**) The histogram represents quantification of the IDEF1 and HMW-IDEF1 band in (**b)** using Image J. Protein levels are expressed as a ratio of IDEF1 or HMW-IDEF1 to HSP80. (**d**) Immunoprecipitation was performed using anti-IDEF1 antibodies on solubilised protein extracts from WT and subjected to immunoblotting with the anti-K48 polyUb antibody Apu2. IB, immunoblotting; IP, immunoprecipitation. The arrow indicates polyubiquitinated IDEF1. Equal protein loading was confirmed by CB (Coomassie Blue stain). Western blot using IDEF1 antibody was used as a loading control for the input of the IP. HC, heavy chain; HMW, high molecular weight. Error bars indicate SD (n = 3). Asterisks indicate P < 0.05 (*) and P < 0.01 (**) in Student’s t test analysis.

There are two main pathways for protein degradation: proteasome-mediated degradation and endocytosis or autophagy-mediated vacuolar degradation^35^. A recent report indicates that IDEF1 is degraded in a 26S proteasome-dependent manner and IBP1.1 (IDEF1-binding protein) prevents its degradation^34^. We analysed the degradation pathway of IDEF1 both in rice tissues and suspension cells by employing the effect of MG132 on the accumulation of IDEF1 protein. After treatment with MG132, a 26S proteasome inhibitor, both roots and leaves accumulated high-molecular-weight IDEF1 (Fig. 6a). We noticed that the initial amounts of unmodified IDEF1 differed dramatically between roots and shoots, although in both tissues the MG132 treatment resulted in IDEF1 accumulation (Fig. 6a). Similar results were also observed by using rice suspension cells (Fig. 6b), and the HMW-IDEF1 level was higher after 20 h of MG132 treatment than after 12 h treatment (Fig. 6c). These results confirm that the IDEF1 degradation occurs via 26S proteasome and is blocked by MG132. Proteins targeted for 26S proteasome-mediated degradation were reported to be marked with a polyubiquitin chain linked through Lys48^36^. A comparable high molecular weight smear was observed when using a K48 polyubiquitin chain-specific Apu2 antibody, highlighting the fact that IDEF1 is also modified by K48-linked ubiquitin chains (Fig. 6d). These results demonstrate ubiquitination affects IDEF1 protein stability as a result of the 26S proteasome-mediated degradation pathway.

Impairment of the 26S proteasome by MG132 treatment resulted in accumulation of both native and ubiquitinated IDEF1 in suspension cells (Fig. 6a) and in shoots and roots of seedlings (Fig. 6b). Similar to pharmaceutical treatment, the amounts of native and ubiquitinated IDEF1 also increased in an antisense line (A9, A20) (Fig. 5a). Therefore, 26S proteasome activity was restored in antisense lines and this resulted in remarkably increased amounts of native and ubiquitinated IDEF1. Even though the main role of MG132 treatment and *CSN6* depletion is to inhibit the polyubiquitinated IDEF1 from entering the 26S proteasome pathway, the increase of native IDEF1 is same to previous reports^34^.

### Transgenic lines show diverse chlorophyll content and metal concentration in response to iron condition

In Arabidopsis, introducing 35S promoter-driven sense and antisense At*CSN6A* cDNA into wild-type results in multifaceted developmental defects^13^. We thus explored if corresponding phenotypes were found in *CSN6* sense and antisense transgenic rice lines and subsequent change in response to different iron condition.

**Figure 7:**
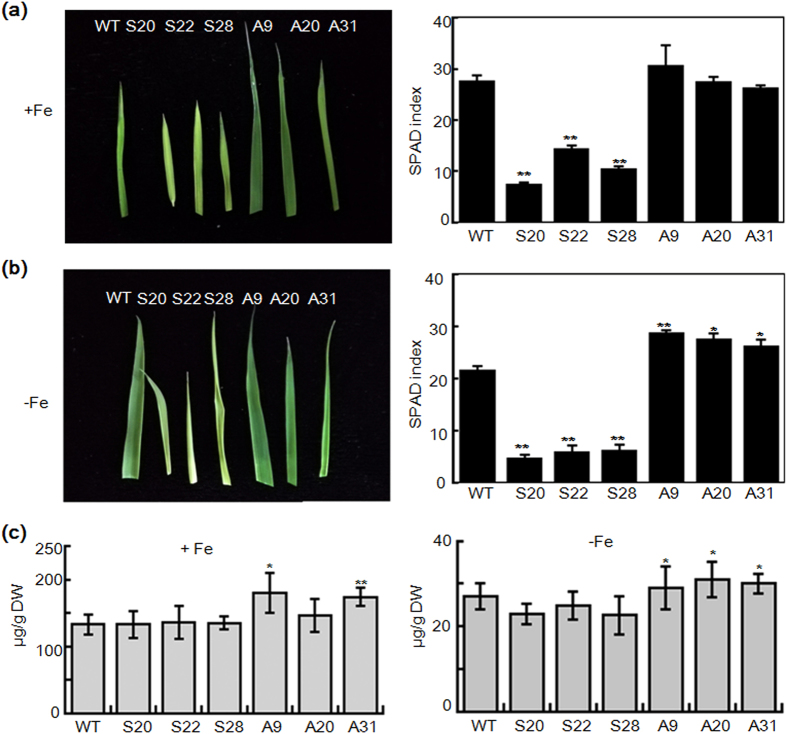
Soil and Plant Analyzer Development (SPAD) index in largest leaves and Fe concentration in seedlings of WT, antisense, and sense lines of 21-day-seedlings. (**a**) Leaf phenotype (left) and chlorophyll content (right) of WT and transformants under normal conditions (+Fe, 100 μM Fe). (**b**) Leaf phenotype (left) and chlorophyll content (right) of WT and transformants under -Fe conditions (0 μM Fe). Bars represent means ± standard error of three independent plants (n = 3). Significant differences from WT were determined by Student’s t-test, P < 0.05 (*), P < 0.01 (**). (**c**) Fe concentration of WT and transgenic rice seedlings in iron sufficient (+Fe, 100 μM Fe) and deficient (0 μM Fe) conditions. S represents T3 seedlings of sense transgenic lines. A represents T3 antisense seedlings of transgenic lines. DW, dry weight. Bars represent the means ± standard errors of three independent analyses (n = 3). Significant differences from WT were determined by Student’s t test, P < 0.05 (*), P < 0.01 (**).

As IDEF1 was repressed (Fig. 5a), sense lines showed lower chlorophyll content (Fig. 7a,b) irrespective of iron condition. This phenotype is similar to IDEF1 knock-down rice during the early stage of Fe deficiency^29^. It suggests sense lines may have lost the factors need to detect iron condition. However, the iron concentration in sense lines did not change (Fig. 7c). In previous work, the I2p-*IDEF1* plants (*IDEF1* gene introduced into rice under the control of the iron deficiency-inducible *IDS2* promoter) grown in an iron deficient hydroponic culture or calcareous soil grew better than non-transformants (NT) plants and contained similar iron concentrations to those in NT plants^23^. They proposed the induced expression of *IDEF1* in rice plants confers tolerance of iron deficiency, most likely through improved iron utilisation within the plant body rather than enhanced iron uptake from the rhizosphere^23^. Therefore, the *CSN6* sense lines had lower iron utilisation and showed reduced chlorophyll content but had no change in iron concentration because of repressed IDEF1.

In contrast, antisense lines (*CSN6* mutant) responded to iron condition. The accumulation of IDEF1 protein and iron-regulated genes in antisense lines (Fig. 5) helped the lines to tolerate iron deficiency. Chlorophyll content of leaves in antisense lines was higher than both WT and sense lines in -Fe condition (Fig. 7b), similar to previous reports^23^,^29^. In addition, antisense lines (A9, A31) in iron sufficient and deficient conditions contained higher iron concentration compared to WT and sense lines (Fig. 7c). Other metal concentrations under both iron conditions did not change significantly in the lines (Fig. S1).

CSN-mediated derubylation of the cullin subunit is important for the progress of proteasome-mediated regulation and a growing number of proteins have been shown to be involved in the proteasome-regulation degradation pathway. We presume that proteins involved in iron uptake may accumulate due to restored degradation in antisense lines and result in different iron concentrations compared to I2p-*IDEF1* plants. All together, these results validate that CSN6 responds to iron condition by regulating the degradation of IDEF1.

## Discussion

In this work, we report the connection between CSN6 down-regulation and IDEF1 accumulation in the early stage of -Fe. Our functional studies reveal that CSN plays a critical role in response to early stage of -Fe.

CSN serves an essential role in a myriad of cellular processes by reversing rubylation through the isopeptidase activity of its CSN5 subunit. CSN5, designated as the derubylation catalytic centre of the complex, is reported to associate with the holocomplex via CSN6^12^. Evidence has shown that CSN6 serves as a molecular platform between protein degradation and signal transduction^37^. Here, we found that CSN6 is responsive to -Fe signals, leading to down-regulation of CSN6 transcription and then protein expression at the early stage of -Fe, while not responsive to -Zn signals (Fig. 1). Although various metabolites affected by Fe availability are thought to influence -Fe responses, the direct binding of Fe to transcriptional regulators is the primary Fe sensing event in plant cells^38^. For example, upon binding iron at haemerythrin (HHE) domains, BRUTUS (BTS) is destabilised and this destabilisation relies on specific residues within the HHE domains^39^. The transcription factor IDEF1 can regulate downstream genes depending on the signal from whether it is binding iron or not. Thus it is interesting to find a factor that is vital to regulate the stability of IDEF1.

CSN5 maintained a constant mRNA level in respond to -Fe (Fig. 2a) and its protein level was down-regulated in day one of -Fe, corresponding to CSN6 (Fig. 2b). As the native band of CUL1 decreased in the first day of -Fe (Fig. 2e), the derubylation activity of CSN declined. The MLN4924 treatment inhibited rubylation conjugation with CUL1 in rice at 35 μM (Fig. 2f), thereby confirming the rubylated state of CUL1. Moreover, the antisense lines had reduced derubylation activity, due to a decline in the native band of CUL1 (Fig. 4e). At the same time, the antisense lines also had reduced CSN6 (Fig. 4c) and CSN5 (Fig. 4e) expression. Therefore, down-regulation of CSN6 impairs the derubylation function of CSN via a decrease in the abundance of CSN5 and then protects the protein from proteosome-dependent degradation. This result is in accordance with previous reports in Arabidopsis^13^. As the COP9 signalosome regulates multifaceted developmental processes through its involvement in ubiquitin/proteasome-mediated protein degradation, it is possible that other proteins related to iron accumulation are involved in 26S degradation.

**Figure 8:**
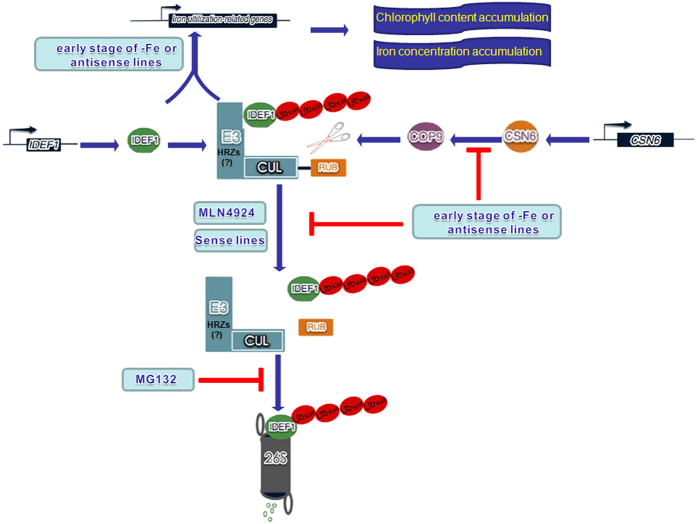
A working model describing the link between CSN6, CUL1, IDEF1, and iron nutrition in rice. On the early stage of -Fe or in *CSN6* antisense lines, *CSN6* expression is down-regulated. Reduced CSN6 leads to reduced expression of CSN5 and COP9 activity (derubylation of CRL), thus retarding the proteasomal degradation of IDEF1, leading to the accumulation of HMW-IDEF1 protein. Elevated IDEF1 up-regulates iron utilisation-related genes such as *IRO2*, *IRT1*, *YSL15,* and *NAS*s, contributing to chlorophyll and iron concentration accumulation. On the later days of -Fe or in sense *CSN6* lines, the derubylation activity of CSN is reactivated as a result of increased *CSN6* transcription. The excess IDEF1 protein is degraded via proteasome-mediated degradation. Treatment with MG132 leads to the accumulation of IDEF1, reminiscent of antisense lines, and the HMW-IDEF1 is linked to K48-polyubiquitin chains. MLN4924 can suppress the rubylation of CUL1 in rice. HRZs are guessed to be the F-box (E3) involved in IDEF1 degradation.

Studies in Arabidopsis indicate that the transcriptional activity of FIT is regulated by proteasome-mediated degradation during -Fe^32^,^40^,^41^. Therefore, FIT is dually regulated by transcriptional and post-transcriptional regulatory mechanisms, and proteasome activity is necessary for the FIT protein to maintain rapid turnover^32^. Similar to Arabidopsis, the IDEF1 protein in rice is degraded in a 26S proteasome-dependent manner and this degradation is prevented by IBP1.1^34^. Here, in *CSN6* antisense lines, IDEF1 degradation is restored and ubiquitinated IDEF1 is accumulated (Fig. 5a). Immunoprecipitation using an anti-IDEF1 antibody and then detection using an anti-FK1 antibody illustrates that IDEF1 is decorated with polyubiquitinations (Fig. 5b). This result is further confirmed by treating with MG132. HMW-IDEF1 responds strongly and rapidly to MG132 treatment in rice seedlings (Fig. 6a) and rice suspension cells (Fig. 6b). These HMW-IDEF1 bands are detected by an anti-K48 ubiquitin antibody and illustrate that IDEF1 is decorated with K48 polyubiquitin chains (Fig. 6d). Therefore, IDEF1 is linked with K48-linked polyubiquitin and targeted to the 26S proteasome for degradation. The transcription factor IDEF1 plays a crucial role in regulating the early response to -Fe in rice. When the -Fe stress occurs, IDEF1 senses the stress signal at an early stage and binds to the Fe deficiency-responsive *cis*-element IDE1^23^, and then up-regulates various Fe uptake/utilisation-related genes, including *IRO2*, *IRT1*, *YSL15*, and *NAS*s. During subsequent Fe-deficiency stages, IDEF1 regulates several Fe deficiency-inducible late embryogenesis enriched genes^18^. Here, the up-regulated genes in CSN6 antisense lines during normal condition are also up-regulated like the genes in WT during early stages of -Fe (Fig. 5c). It was suggested that the CSN6 may sense the -Fe signal and then regulate IDEF1 degradation. Considering these studies, we suggest a model for the regulation of IDEF1 stability by CSN6 during the first stage of -Fe in rice (Fig. 8). On the early stage of -Fe or in the antisense lines, the derubylation activity of CSN is decreased due to down-regulation of *CSN6* transcription. Less CSN increases CRLs ubiquitin activity and repairs proteasome-mediated degradation, thus leads to increased ubiquitinated IDEF1 and native IDEF1 after de-ubiquitination. The elevated levels of IDEF1 and the polyubiquitinated form of IDEF1 up-regulate downstream Fe utilisation-related genes and lead to chlorophyll content and iron concentration accumulation (Figs 7b,c and 8). However, in the following days of -Fe or in the sense lines, the derubylation activity of CSN is reactivated as a result of increased CSN6 transcription. The excess IDEF1 protein is degraded via proteasome-mediated degradation. MG132 could inhibit this process (Figs 6a and 8). However, what type of E3 is specific to IDEF1 is still unclear. As we know, certain E3s ubiquitinate specific substrates in response to environmental changes and are therefore important in stress responses^42^,^43^. Recent data show that IRT1 Degradation Factor 1, a RING E3 Ubiquitin Ligase, regulates the degradation of IRT1 in Arabidopsis^44^. Another result found that the genes possessing ubiquitination activity, which are designated in *Oryza sativa* as Haemerythrin motif-containing Really Interesting New Gene (RING)-and Zinc-finger proteins (OsHRZs), are potent negative regulators of -Fe response^45^. Kobayashi *et al*. (2013) reports OsHRZs shows some functional similarity with human FBXL5, because the latter contains an F-box domain, and OsHRZs knockdown plants exhibit substantial tolerance to iron deficiency and accumulate more iron in their shoots and grains irrespective of soil iron conditions^45^. The phenotype of OsHRZs knockdown plants looks like *CSN6* antisense lines. Thus, we suppose the HRZs may act as an F-box to regulate the degradation of IDEF1 and have added it in model (Fig. 8). Therefore, the ubiquitination plays a critical role in the stability and function of the transcription factor or its transport. Indeed, iron-induced ubiquitination regulations have already been involved in the pathway responding to iron stress signals^45^. Further investigation is required to focus on the specific modulator that regulates IDEF1 degradation in different iron conditions.

We present a certain link between CSN6 (COP9 signalosome subunit 6), cullin1, IDEF1, and iron nutrition. CSN6 can affect the proteasome-mediated degradation of IDEF1 by regulating the function of the COP9 signalosome. Due to down-regulated CSN6 and then CSN activity during the early stage of -Fe, IDEF1 accumulates in rice. The accumulation of IDEF1 protein is then necessary to help overcome the iron deficiency stress via increasing the expression levels of iron uptake/utilisation-related genes in rice. This provides a new sight on iron homeostasis in the early stage of -Fe, based on the upstream regulation of IDEF1.

## Methods

### Construction of sense and antisense lines of *CSN6* transgenic rice

The coding region of *CSN6* was amplified from a rice cDNA library using the following primers: FL, 5′-ATACCATGGCGCGACTCCGACGATGTC-3′; RL, 5′-ATACCATGGCATGAAAGCACCTCGGCC-3′, which introduced *Spe*I sites at both ends. The coding region of rice *CSN6* was cloned into pCAMBIA1302 in either the sense or antisense orientation under the control of the 35S promoter, in which the target proteins were fused to mGFP. Sense and antisense recombinants were screened by *Sal*I digestion. The expression vectors were transformed into *Agrobacterium* EHA105 using the freeze-thaw method^46^. The constructs were transformed into rice callus using an *Agrobacterium*-mediated method^46^. This resulted in the expression of the CSN6::mGFP fusion protein in sense lines, and reduced the expression of endogenous *OsCSN6* in antisense lines. Seventy-seven independent resistant lines were obtained for both the sense and antisense constructs in the T1 generation. The transgenic seeds were identified by hygromycin resistance (50 mg/L hygromycin) (CALBIOCHEM, Germany). Out of the 45 T1 sense rice lines tested, 27 were positive for the transgene; 28 out of 32 antisense lines were positive for the transgene. T2 and T3 plants were harvested from individual plants, and were examined by both hygromycin resistance and real-time PCR assay. We chose three sense and three antisense lines of T3 generation with significantly altered *CSN6* levels for further examination.

### Plant material and growth conditions and inhibitor treatment

#### Rice seedling growth condition

Both wild-type (WT) rice (*Oryza sativa* L. cv. *Japonica*) and transgenic rice were used for growth and expression assays. Rice seeds were washed with sterilised distilled water and sterilised with 2% NaClO for 30 min, then washed seven times in sterilised water. T3 seeds of *CSN6* sense and antisense lines were germinated on MS solution containing hygromycin B (50 mg L^−1^) (full nutrition, 100 μm Fe). WT seeds were germinated on MS solution lacking hygromycin B. The seeds were cultured 14 days under 16 h light/18 h dark and 25 °C (14-day-old seedlings). The 14-day-old seedlings were exposed to iron deficiency (-Fe) treatment by transferring seedlings into MS solution without Fe-EDTA. The nutrient solution was renewed every two days. For time course experiments, roots or seedlings were harvested from at least three plants of each line, to reduce biological variance, and were then used for expression analysis.

#### Rice suspension cell culture

Establishment of cell suspension cultures was based upon the method of Ozawa and Komamine^47^. The rice seeds were sterilised in 10% sodium hypochlorite for 30 min. After three rinses with sterilised water, the seeds were placed on solid N6 medium supplemented with 2,4-D (2 mg/L), proline (10 mM), casein hydrolysate (300 mg/L), sucrose (30 g/L) (N6D), and Gelrite (4 g/L). The cultures were incubated at 25 °C using a 16 h light (50 μmol/m·s) and 8 h dark cycle. Calli (2-weeks old) derived from seed were transferred to 100 mL Erlenmeyer flasks that contained 15 mL of liquid N6D media. Cell clusters were subcultured by transferring 0.2 mL (packed cell volume) into 15 mL fresh media in 100 mL Erlenmeyer flasks every three days. The cultures were incubated at 28 °C on a gyratory shaker (90 rpm) in the dark.

#### Inhibitor treatment

For inhibitor studies (MG132, Sigma-Aldrich, MO63103 USA), 14-day-old seedlings were transferred to liquid MS full nutrition (+Fe) solution with MG132 (40 μM) and DMSO after 12 h and 20 h. After three days suspension cells were transferred to fresh N6D medium with MG132 (40 μM) and DMSO after 12 h and 20 h. 14-day-old seedlings were transferred to liquid MS full nutrition (+Fe) solution with MLN4924 (0 μM, 15 μM, 25 μM, 35 μM, 50 μM) (ApexBio, USA) and DMSO after seven days. MLN4924-treated materials is washed three times in sterilised water and used for further studies.

### RNA isolation and qRT-PCR analysis

Total RNA was isolated from each rice seedling using the NucleoSpin RNA Plant Kit (Macherey-Nagel, Germany). For cDNA synthesis, we used 2 μg of total RNA as a template and PrimeScript^TM^ 1st strand cDNA Synthesis Kit (Takara Shuzo) in a 20 μL reaction mixture. Quantitative real-time PCR (qPCR) was performed with a Takara SYBR Premix Ex Taq Kit (Takara Shuzo). *Actin1* mRNA levels were used to normalise the expression of each gene. Changes in expression were calculated using the ΔΔCt method. The gene-specific primers used are listed in Supplementary Table S1.

### Protein extraction and immunoblotting

Total protein was extracted from three-day-old rice suspension cells and 14-day-old rice tissue of WT or *CSN6* transgenic rice grown in the conditions described above. Collected suspension cells and rice tissues were ground in liquid nitrogen and resuspended in RIPA buffer^48^ (50 mM Tris-HCl pH 7.5, 150 mM NaCl, 0.5% sodium deoxycholate, 1% Nonidet P-40, 0.1% SDS, and protease inhibitor cocktail mixture). After centrifugation at 15,000 × *g* for 15 min at 4 °C, total protein was collected, and prepared for western blotting. Proteins were separated by SDS-PAGE and transferred to a PVDF (polyvinylidene difluoride) membrane by electroblotting. Western blots were processed using appropriate primary antibodies (Supplementary Table S2), and were then incubated with the appropriate HRP-linked secondary antibodies. ECL prime reagents were acquired from GE Healthcare Life Science. Chemiluminescence was accurately quantified using the LAS3000 Imaging System.

### Immunoprecipitation

About 300 μL total protein was obtained from the procedures described above and supernatant was incubated with 3 μL anti-IDEF1 antibody for 4 h at 4 °C on a rotating wheel. The samples were then incubated with 20 μL Protein G Sepharose 4 Fast Flow (GE, 17-0618-01) for 3 h at 4 °C on a rotating wheel. Before the incubation, beads were washed with 500 μL PBS three times. The IDEF1-IgG complexes were eluted from the beads by adding 48 μL RIPA, then with 10 μL 5 × SDS loading buffer and heated at 95 °C for 6 min. The acquired proteins were then used for immunoblotting.

### Measurements of chlorophyll content and metal concentration in rice seedlings

A SPAD-502 chlorophyll meter was utilised to measure chlorophyll content^23^. All of the data underwent statistical analysis. Rice seedling metal concentration analysis was performed according to Han *et al*.^49^ and Tan *et al*.^50^. The seedlings were cleaned and divided in 3 piles of 10 g each and the dried seedlings were weighed. The seedlings were placed into nitrification tanks and treated with nitric acid, washed three times in water, and then dried in an oven at a temperature of 80 °C. Nitric acid and water in the ratio of 4:1 was injected into the oven. After the nitration process, the digestive juices were transferred into a 25 mL flask. The liquid was measured by inductively coupled plasma mass spectrometry (ICP-MS, USA) using the PerkinElmerOptima 2000 DV as the ICP-MS instrument. The experiment was repeated three times and all of the data were statistically analysed.

### Locus Numbers

Sequence date from this article can be found in the GenBank under locus numbers AEX88465 (IDEF1), NP_001054112.1 (CSN5), CAJ81251.1 (CSN6), and BAD61452.1 (CUL1).

### Significance

The transcription factor IDEF1 upregulates various Fe uptake/utilisation-related genes during the early stage of Fe deficiency in rice. However, how the IDEF1 is regulated under Fe deficiency condition still unknown. We address a hypotheses, namely, a certain link between CSN6 (COP9 signalosome subunit 6), cullin1, IDEF1 and iron nutrition. We reveal that the accumulation of IDEF1 protein regulated by CSN6 under -Fe, is necessary to help overcome the iron deficiency stress in rice. The main conclusion of the manuscript is important and timely, because CSN6 can affect the proteasome-mediated degradation of IDEF1 with K48 polyubiquitin chain, thus the early Fe-deficiency response in rice, by regulating the function of the COP9 signalsome. It provides a new sight on iron homeostasis in the early stage of Fe deficiency, based on the upstream of IDEF1.

## Additional Information

**How to cite this article**: Tan, S. *et al.* CSN6, a subunit of the COP9 signalosome, is involved in early response to iron deficiency in *Oryza sativa*. *Sci. Rep.* **6**, 25485; doi: 10.1038/srep25485 (2016).

## Supplementary Material

Supplementary Information
