# Supplementary material for: CSN6, a subunit of the COP9 signalosome, is involved in early response to iron deficiency in Oryza sativa
Source: Sci Rep. 2016 May 3;6:25485. doi: 10.1038/srep25485 (PMC4853791; doi:10.1038/srep25485)
Supplement: Supplementary Information [file srep25485-s1.doc]

CSN6, a subunit of the COP9 signalosome, is involved in early response to iron deficiency in *Oryza sativa*

Song Tan^†1^, Fang Liu^†1^, Xiao-Xi Pan^1^, Yue-Peng Zang^1^, Fei Jin^1^, Wei-Xi Zu^1^, Xiao-Ting Qi^1^, Wei Xiao*^1,2^, Li-Ping Yin*^1^

^1^College of Life Science, Capital Normal University, Beijing 100048, China

^2^Department of Microbiology and Immunology, University of Saskatchewan, Saskatoon, SK, S7N 5E5, Canada

**Address Correspondence to:**

[yinlp@cnu.edu.cn](mailto:yinlp@cnu.edu.cn) and [wei.xiao@usask.ca](mailto:wei.xiao@usask.ca)

Tel: 86 (0) 10 68901692

**Supplementary Figures and Tables**

**The following materials are available in the online version of this article:**

Figure S1. Metal content of WT and transgenic rice seedlings in iron sufficient (100µM Fe) (a) and deficiency (0µM Fe) (b) condition.

Table S1. Primers used in this work

Table S2. Primary antibodies used in this work


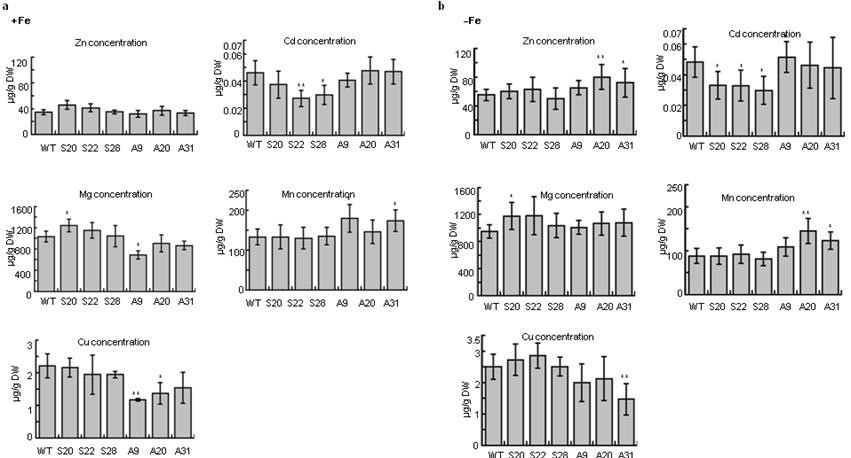


Figure S1: Metal content of WT and transgenic rice seedlings in iron sufficient (+Fe, 100 µM Fe) (a) and deficiency (-Fe) (b) condition.

S represents T3 seedlings of sense transgenic lines. A represents T3 antisense seedlings of transgenic lines. Bars represent the means ± standard errors of three independent analyses (n = 3).

Significant differences from WT were determined by Student’s t test,*P<0.05, *P<0.01.

Supplementary Tables

**Table S1. Primers used in this work**

| Gene name | Forward primer | Reverse primer |
| --- | --- | --- |
| *Actin* | CAGCACATTCCAGCAGAT | GGCTTAGGATTCTTGGGT |
| *CSN6* | ACTTCTATGTGCTGGGTTGG | TTACAGGGAGATCCTTCTGG |
| *IDEF1* | GCCATTCATCAGCAGACAAC | CTTCTCCTGGATTGCCAACT |
| *IRO2* | GGAAGAAGAGGAGAAGAGGAGAT | CCAAGCCAGACCAGTAGTAGTA |
| *IRT1* | ATCACACTCTGCGGCATCAT | GAACAACCACGCTACAACACT |
| *YSL15* | TGGAACAAGATGAACAAGAAGGAG | TAGCGAGAGCAAGGATAGAAGAA |
| *NAS1* | GTCTAACAGCCGGACGATCGAAAGG | TTTCTCACTGTCATACACAGATGGC |
| *NAS2* | TGAGTGCGTGCATAGTAATCCTGGC | CAGACGGTCACAAACACCTCTTGC |
| *CSN5* | CCCTACAAGGACTGTTTCTG | AAGTAGATCAAGGAGATGAGAAT |
| *CUL1* | TGTGATGGAATGTGTGGAGC | ACACATTTGGGTTGTCTTTAT |

Table S2 Primary antibodies used in this work

| Primary Antibodies | | | | | |
| --- | --- | --- | --- | --- | --- |
| Name | Host | Dilution | Antigen | Company | Reference |
| GFP | Mouse | 1:500 | 1-238 a.a. | Santa Cruz | De Lucas et al. (2008)^1^ |
| CSN6 | Rabbit | 1:1000 | CNVSDFFTRVKAQAA | Beijing Protein Innovation | The specificity 1:1000 for the anti-CSN6 by our lab |
| AtCSN5 | Rabbit | 1:1000 | Full length protein | Enzo | Gusmaroli et al.(2007)^2^ |
| IDEF1 | Rabbit | 1:1000 | AAVHQQTVPFPNPF*C | Beijing Protein Innovation | The specificity 1:1000 for the anti-IDEF1 by our lab |
| CUL1 | Rabit | 1:1000 | DSASNKKPEKKD*C | Beijing Protein Innovation | Gusmaroli et al.(2007)^2^ |
| HSP80 | Mouse | 1:5000 | Full length protein | Beijing Protein Innovation | Li et al. (2011)^3^ |
| FK1 | Mouse | 1:2500 | Poly-ubiquitinylated-lysozyme | Millipore | Haglund et al. (2003)^4^ |
| K48 | Mouse | 1:2000 | anti K48 polyubiquitin Apu2 | Millipore | Martins et al. (2003)^5^ |

**Reference**

1. De Lucas, M. *et al.* A molecular framework for light and gibberellin control of cell elongation. *Nature* **451**, 480-484 (2008).

2. Gusmaroli, G., Figueroa, P., Serino, G. & Deng, X. W. Role of the MPN subunits in COP9 signalosome assembly and activity, and their regulatory interaction with Arabidopsis Cullin3-based E3 ligases. *Plant Cell* **19**, 564-581 (2007).

3. Li, X. *et al.* Identification and validation of rice reference proteins for western blotting. *J. Exp. Bot.* **62**, 4763-4772 (2011).

4. Haglund, K. *et al.* Multiple monoubiquitination of RTKs is sufficient for their endocytosis and degradation. *Nat. Cell Biol.* **5**, 461-466 (2003).

5. Martins, S. *et al.* Internalization and vacuolar targeting of the brassinosteroid hormone receptor BRI1 are regulated by ubiquitination. *Nat. Commun.* **6**, 7151 (2015).
